# Dysmorphometrics: the modelling of morphological abnormalities

**DOI:** 10.1186/1742-4682-9-5

**Published:** 2012-02-06

**Authors:** Peter Claes, Katleen Daniels, Mark Walters, John Clement, Dirk Vandermeulen, Paul Suetens

**Affiliations:** 1 Department of Electrical Engineering - processing of speech and images - medical image computing, KU Leuven, Herestraat 49 Bus 7003, Leuven, 3000, Belgium; 2 Cranio-Maxillo-Facial Unit, Princess Margaret Hospital for Children, Perth, Australia; 3 Melbourne Dental School, The University of Melbourne, Melbourne, Australia; 4 IBBT-K.U.Leuven Future Health Department, KU Leuven, Leuven, Belgium

**Keywords:** Geometric Morphometrics, Dysmorphometrics, Procrustes ML-estimator, Pinocchio effect, robust statistics, abnormalities, outlier-processes

## Abstract

**Background:**

The study of typical morphological variations using quantitative, morphometric descriptors has always interested biologists in general. However, unusual examples of form, such as abnormalities are often encountered in biomedical sciences. Despite the long history of morphometrics, the means to identify and quantify such unusual form differences remains limited.

**Methods:**

A theoretical concept, called dysmorphometrics, is introduced augmenting current geometric morphometrics with a focus on identifying and modelling form abnormalities. Dysmorphometrics applies the paradigm of detecting form differences as outliers compared to an appropriate norm. To achieve this, the likelihood formulation of landmark superimpositions is extended with outlier processes explicitly introducing a latent variable coding for abnormalities. A tractable solution to this augmented superimposition problem is obtained using Expectation-Maximization. The topography of detected abnormalities is encoded in a dysmorphogram.

**Results:**

We demonstrate the use of dysmorphometrics to measure abrupt changes in time, asymmetry and discordancy in a set of human faces presenting with facial abnormalities.

**Conclusion:**

The results clearly illustrate the unique power to reveal unusual form differences given only normative data with clear applications in both biomedical practice & research.

## Background

Morphometrics involves the measurement of morphology based on quantitative descriptions [1]. Different definitions of form exist but the most commonly adopted is that form is defined as size and shape independent of position and orientation [2]. Morphometric methods are designed to measure form and variations in form and have been extensively used in evolutionary, developmental and systematic biology. In those contexts, they provide information on phylogenetic relationships and the evolutionary development of organisms [3]. They also allow for taxonomic discrimination of sampled populations to test whether these were drawn from different (sub)species or not [4]. However, in biomedical contexts unusual from instances such as abnormalities [5] are often encountered and of interest. For example abnormalities may include deformations in form due to congenital malformation and/or environmental constraints as well as abrupt changes in form associated with traumatic injuries and surgical interventions. The occurrence of an abnormality is more than just variation in form. Consequently, testing for hypothesized "unusual form" represents another type of challenge and is dependent on the definition used. Here, we follow a pattern analysis perspective where an abnormality is defined as a pattern in the data that does not conform to some expected behavior [6].

Morphometric analysis underwent a conceptual revolution during the 1980s and 1990s [7]. As a result, a variety of methods and approaches have evolved with some clear conceptual and technical differences. Continuous boundary representations, such as Fourier descriptors e.g., have been employed to quantify form based on outline data [3,8]. In contrast, geometric morphometrics [9] uses homologous landmarks, these being defined as "a point of correspondence on an object that matches between and within populations" [10]. Within these landmark-based approaches three different ways to deal with the confounders of position and orientation have been proposed [11]: (1) Superimposition [12,13], involving placing the landmark data into a common frame of reference, (2) Deformation [14-17], where form differences are described in terms of deformation fields of one object into another and (3) Linear Distances [18], where all possible distances between landmarks and not their absolute position and orientation are measured. Advances over the past few decades have made morphometric analysis easier, more accessible and therefore more attractive to use in practical studies. For example, with the advent of three-dimensional (3D) tomographic imaging and rapid surface scanning, the quantification of form can now be performed indirectly and virtually both in 2D and 3D [19].

However, none of the existing approaches in morphometrics are particulary well suited to deal with abnormalities as such and hence are of limited use in their measurement. One reason for this is that unusual form differences typically introduce a Pinocchio effect characterized by a substantial, but localized, form change or difference [4]. For example, congenital malformations often have a discontinuous impact on form. A second and more important reason for the limited use of morphometrics, so far, is that there is no real mechanism in place to separate unusual from usual form differences. To address this dilemma, a new theoretical concept and associated modelling methodology, termed dysmorphometrics, is proposed. The topography of form abnormalities is encoded in a dysmorphogram, which is used to visualize and facilitate any subsequent quantification of the detected form differences of interest.

The manuscript is organized as follows: We start with the morphometric background. First, landmark superimposition following a likelihood formulation is shortly introduced. Secondly, the influence of and current solutions to the Pinocchio effect are given. The next section introduces and defines dysmorphometrics and extents the superimposition accordingly to deal with form abnormalities. Furthermore, we illustrate the relationship with the known solutions for the Pinocchio effect. Throughout the results and without loss of generality, we use human faces as example biological forms of interest (Appendix A). We define three different but typical types of questions related to facial form in clinical practice starting from 3D surface scans, e.g., and dysmorphometrics is applied to answer these questions: measuring abrupt form changes, asymmetry, and discordancy of features in human faces presenting abnormalities. A discussion centered on comparisons with current morphometric techniques follows and future possibilities, limitations, and work of dysmorphometrics conclude the article.

## Methods

### Morphometric Background

#### Landmarking

Homologous *landmarks *are fundamental to geometric morphometrics as a biomathematical primitive. They are often defined as precise locations on biological forms that hold some developmental, functional, structural, or evolutionary significance [11]. However, owing to the lack of anatomically discrete features in regions of the face (cheeks and forehead for example), landmarks can only provide a sparse representation of the complete facial form and salient features can be overlooked [20]. Therefore, in addition to using true landmarks, points defined by relative locations (*pseudo-landmarks*), for example "the point of highest curvature", or defined relative to other landmarks (*semi-landmarks *[21]), for example "halfway between the corners of the eyes", can be used as well [19]. Alternatively, a spatially-dense indicated set of landmarks can be obtained using an anthropometric mask and mapping technique [22-24]. The latter provides a spatially-dense set of *quasi-landmark *indications, which are used here and are essentially obtained using a non-rigid surface registration (mapping) of a predefined facial template (anthropometric mask) [25].

Independent of the kind of landmarks used, in order to compare different form instances the same homologous or corresponding landmarks are indicated. Mathematically, a form instance is then represented as a (landmark) configuration consisting of the coordinates of a list of ordered landmarks: *C *= {*l_j_|j *= 1,... *K*} with *K *the number of landmarks and the ordering index *j *defining the unique label of the associated landmark *l_j _*= (*x_j_*, *y_j_*, *z_j_*) in 3D. Hence, a single form is defined as a *K × *3 matrix.

#### Superimposition

When comparing a particular configuration or a group of configurations to another, any and all orientation and/or position differences are considered unimportant. However, both orientation and position influence the actual recorded coordinates. As mentioned previously, several approaches exist to deal with this problem; one of which is superimposition. Superimposition has the advantage of being quite intuitive and easy to visualize. It accounts for the confounding variables related to position and orientation through the optimal transformation of further analysis. In the context of a so called ordinary analysis, given two configurations *C *and $\overset{`}{C}$, differences in form are defined based on the residual spatial differences *D *after superimposition. This can be formally written as:

(1) $$C=T\left(\overset{`}{C},\theta \right)+D$$

with *T*(., *θ*) a transformation model encoded by a set of parameters *θ*, that are reflecting rotation, translation and scale operations (Appendix B). Finding the optimal superimposition requires the estimation of the optimal transformation parameters *θ *such that *D *reflects true differences in form. Following a likelihood formulation of this problem requires a statistical model with an associated distribution that is assumed to have generated the observed data [26]. The likelihood of transformation parameters is expressed as: $L\left(\theta \right)=\text{Pr}\left(C,\overset{`}{C}|\theta \right)=\text{Pr}\left(D|\theta \right)$ and the optimal superimposition is obtained following a Maximum Likelihood (ML)-Estimator:θ^=arg minθl(θ) where *l *(*θ *) is the negative log-likelihood (NLL): *l *(*θ *) = *- *log *L*(*θ *).

In morphometrics the Procrustes ML-estimators from Goodall [27], assuming a Gaussian perturbation model, are the most commonly known and used. Here, the form difference *D *is modeled as a zero-mean matrix of Gaussian displacements *D ≈ N_K _*_× 3 _(0, Ω). In its most general form, local perturbations for each landmark may be unequal and may even be correlated with each other as specified by the elements in the covariance matrix Ω. According to Goodall [27] this covariance or so-called *model metric *accommodates two sources of variation in the observed data: measurement error and variation in shape.

As a *superimposition metric *the covariance can be user-defined or may be set to an estimate of the model metric. In the latter case any unknown parameters in the model metric are to be estimated on top of the unknown transformation parameters. This can prove to be challenging and a series of simplifications is therefore typically introduced: (a) under the assumption that the directions of variation are the same between different landmarks the full covariance matrix can be factorized using the Kronecker product Ω = ∑ ⊗ Ξ. ∑ is a *K × K *covariance matrix for the rows of *D *reflecting variances and correlations among landmarks. Ξ is a 3 *× *3 covariance matrix for the columns of *D *or the dimensions and *D ≈ N_K_*_,3 _(0, ∑, Ξ). (b) Under the assumption that the displacements around a landmark are isotropic the dimension covariance matrix becomes the identity matrix *D ≈ N_K_*_,3 _(0, ∑, I_3_). (c) Under the assumption that landmark differences are independent, the landmark covariance matrix is a diagonal matrix and (d) under the assumption that the landmark differences are identically distributed (i.e. they are homoscedastic) we have *D ≈ N_K_*_,3 _(0, *σ*^2^I *_K_*, I_3_). Because of the independency assumption, the probability *Pr *(*D| θ *) can be factorized into a product of individual landmark displacement (*d_j_*) probabilities $\overset{K}{\underset{j=1}{\prod }}Pr\left(d_{j}|\theta \right)$ (e) Finally, under the assumption that the distribution is the standard normal distribution we have *D ≈ N_K_*_,3 _(0, I *_K_*, I_3_). This results in the following series of NLL simplifications:

(2) $$l{\left(\theta \right)}_{\left(a\right)}=\frac{1}{2}{∥T\left(\overset{`}{C},\theta \right)-C∥}_{\Sigma^{-1},\Xi^{-1}}^{2}+\frac{K}{2}\text{log}|\Xi |+\frac{3}{2}log|\Sigma |$$

(3) $$l{\left(\theta \right)}_{\left(a,b\right)}=\frac{1}{2}{∥T\left(\overset{`}{C},\theta \right)-C∥}_{\Sigma^{-1},I_{3}}^{2}+\frac{3}{2}log|\Sigma |$$

(4) $$l{\left(\theta \right)}_{\left(a,b,c\right)}=\overset{K}{\underset{j=1}{\sum }}\left(\frac{1}{2\sigma_{j}^{2}}{∥T\left({\overset{`}{l}}_{j},\theta \right)-l_{j}∥}^{2}+log\sqrt{2\pi }\sigma_{j}\right)$$

(5) $$l{\left(\theta \right)}_{\left(a,b,c,d\right)}=\overset{K}{\underset{j=1}{\sum }}\left(\frac{1}{2\sigma^{2}}{∥T\left({\overset{`}{l}}_{j},\theta \right)-l_{j}∥}^{2}+log\sqrt{2\pi }\sigma \right)$$

(6) $$l{\left(\theta \right)}_{\left(a,b,c,d,e\right)}=\overset{K}{\underset{j=1}{\sum }}{∥T\left({\overset{`}{l}}_{j},\theta \right)-l_{j}∥}^{2}$$

Note that any constant term (independent from all parameters) in these simplifications has been omitted as they do not influence the ML-estimation. Also note that the most simplified version with the NLL given in (6) is the frequently used Least Sum of Squares (LSS) superimposition [12].

The Procrustes solutions to the superimposition problem are desirable in shape analysis because of the straightforward link between form differences as squared residual errors and variances. This linkage with conventional multivariate statistics is known as the *Morphometric Synthesis *[28]. The Procrustes ML-estimators are also used when comparing more than two configurations simultaneously in a generalized setup (introducing an additional sum over the different configurations in the NLL's). In this generalized analysis different configurations are superimposed to a consensus configuration, typically an estimated geometric mean.

#### The Pinocchio effect

**Figure 1:**
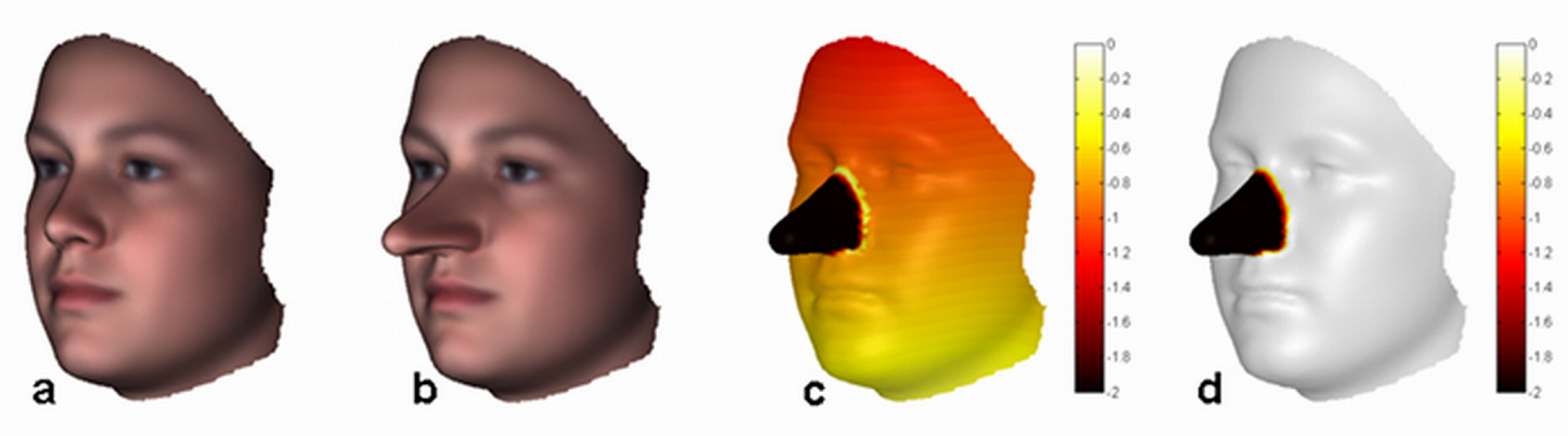
**The Pinocchio effect**. The Pinocchio effect known in shape analysis, is the large change of limited features or landmarks in an object or organism. (a) Pinocchio honest. (b) Pinocchio lying. The tip of the Nose grows forward by an amount of 30 mm. (c) Local landmark superimposition differences after a LSS superimposition or Procrustes-fit of (b) onto (a). The color-scale ranges from 0 mm (white) to 2 mm (dark red) to more than 2 mm (black) difference. Note the smearing out effect of the 'affected' landmarks in the nose onto the 'unaffected' landmarks on the rest of the face after superimposition. (d) Same after robust superimposition of (b) onto (a). Note the perfect alignment of 'unaffected' landmarks. (d) Also depicts the dysmorphogram using a color-scale such that everything except white reflects outliers to some degree with black being the strongest outliers.

In order to illustrate the challenge in studying form abnormalities we use the well-known Pinocchio effect in shape analysis [4]. This effect is characterized by a substantial, but localized, form change or difference, as depicted in Figure 1. The Pinocchio effect influences a Procrustes superimposition using (6), e.g., between the honest (Figure 1(a)) and lying state (Figure 1(b)) of Pinocchio. The large differences of the 'affected' nose landmarks are contaminating the optimal placement of the 'unaffected' remaining landmarks as well. As a result all the landmarks are improperly aligned (Figure 1(c)) and any residual differences do not reflect true differences in form. An analysis of the Pinocchio effect can be obtained by casting the superimposition into an M-estimator formulation:

(7) $$\hat{\theta }=argmin_{\theta }\overset{K}{\underset{j=1}{\sum }}\rho \left(d_{j}\right)$$

**Figure 2:**
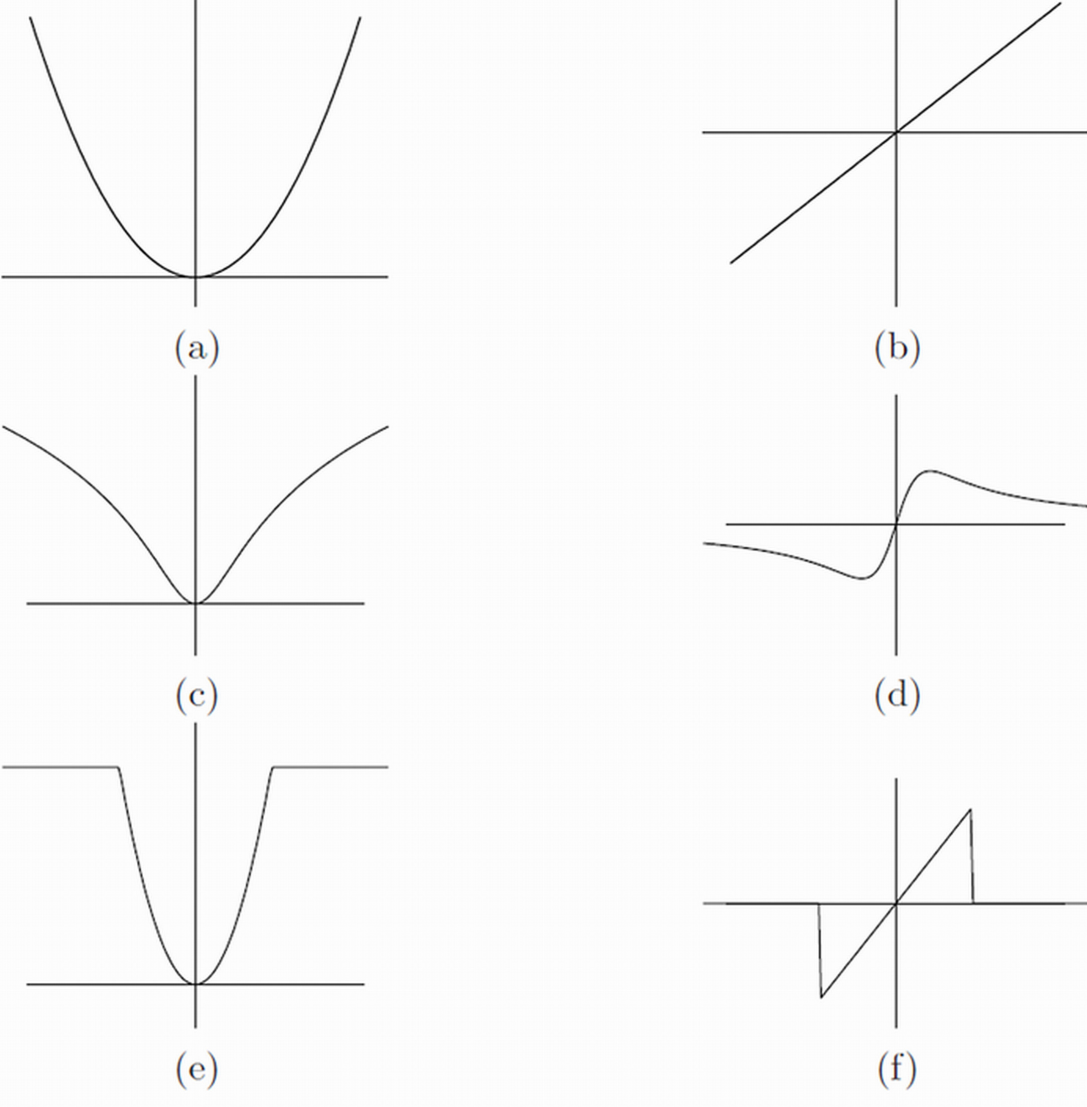
**M-estimators**. Different M-estimators (left column) and their outlier influence functions (right column). (a,b) The Quadratic M-estimator, (c,d) The Lorentzian estimator and (e,f) The Truncated Quadratic. The original Quadratic has a linear increasing outlier influence without bound, while both the Lorentzian and the Truncated Quadratic have interesting saturating properties.

The *ρ*-function is a loss function having a unique minimum when the residual error $d_{j}=∥T\left({\overset{`}{l}}_{j},\theta \right)-l_{j}∥$ is zero. The NLL in (6) for example is equivalent to using a Quadratic M-estimator with a *ρ*-function defined as *ρ*(*x*) = *x*^2 ^and illustrated in Figure 2(a). An analysis of the Pinocchio effect is then performed by examining the equivalent M-estimator through its influence function [29], which characterizes the bias that a particular measurement has on the solution and is proportional to the derivative of the *ρ*-function. For the Quadratic M-estimator the influence function is *ψ*(*x*) = *x *which is depicted in Figure 2(b). It can be seen that the influence of substantial form differences increases linearly and without bound, as expected.

A robust alignment, not influenced by the Pinocchio effect, is illustrated in Figure 1(d), which can only be obtained using an appropriately robust superimposition and associated estimator. Chronologically, the first such robust technique in morphometrics was suggested in [13] and is the so-called resistant-fit using a repeated median (RM) estimator. However, this technique fails to be successful in practice as it exhibits a high time complexity whilst convergence behavior is unclear and dependent on the initial alignment [10]. Another robust median-based estimator is the Least Median of Squares (LMedS) estimator [30], where the sum in (7) is simply replaced by the median.

Alternatively, as suggested by [10], any type of robust estimator can be used such as, for example, robust M-estimators for which a wide range of possible choices has been suggested within the field of robust statistics. Good choices are the redescending M-estimators that have an influence function that is increasing near the origin but decreasing towards zero again further away from it. Two such examples are the Lorentzian and the Truncated Quadratic (TQ) estimator whose *ρ*- and influence functions are illustrated in Figure 2(c,d) and 2(e,f) respectively. They both completely ignore gross form differences during superimposition but do not completely ignore moderately large differences as the median approaches do. Note that different M-estimators imply different underlying distributions that are not necessarily the same as the Gaussian distribution used in Procrustes ML-estimators.

Only recently, tractable and constrained solutions to the model metric estimation for heteroscedastic Gaussian distributed landmarks were formulated allowing for the modelling of individual variances per landmark. Essentially, the model metric can be adapted to allow for larger mobility (higher variance) in the affected nose landmarks and lower mobility (lower variance) in the invariant substructure or unaffected landmarks. The result is a variance weighted LSS solution. In [31] the NLL in (4) without the log term is used and is theoretically linked with a scaled mixture model of Gaussians enabling "large-scale" tolerant superimposition. Indeed, a scaled mixture of Gaussians is a Heavy-tailed distribution known to provide robustness. The shape of the Heavy-tailed distribution is dependent on the prior distribution constraining the variance estimations. In the case of an inverse gamma prior distribution, e.g., the Student T-model is obtained [31]. Similarly, but within a generalized analysis, a complete covariance model metric estimation also allowing for landmark dependency or correlation (using the NLL in (2)) is given in [26].

### Dysmorphometric Extensions

#### Motivation: The Pinocchio dilemma

Morphometrics is primarily focused on form related questions such as: What is the average form and what are the patterns of variation around it within a population, or how do groups differ in shape and what is the functional importance of those differences [9]? The morphometric dilemma raised by the Pinocchio effect is whether to include or exclude this case completely or partially from the study, as the morphometric approach studies only typical variation and co-variation over all the observed landmarks in populations and not any atypical localized differences. If Pinocchio is unique within a population sample, the strength of his uniqueness will influence the analysis of typical variability in the population. For the model metric estimation of a population with heteroscedastically distributed substructures, e.g., a higher variance around the nose is estimated because of Pinocchio. In other words, the model metric is stretched to include Pinocchio and this inclusion might not be desirable. One possible course of action is to use a robust procedure to superimpose first, analyze the residuals and to ignore suspicious landmarks in a subsequent Procrustes analysis as missing data [10].

Dysmorphometrics, in contrast to morphometrics, is focused on questions such as: What makes an individual different and what are the patterns of variation of that particular difference over a group of individuals? For example, it strives to provide an answer to the question 'what specific feature makes Pinocchio different?' In such a context, the only differences of interest are the 'affected' landmarks and a robust superimposition as in Figure 1(d) is preferred, if not crucial. Stated differently, in morphometrics, typical (co-)variances are the variables of interest and the atypical variations are the 'nuisance' variables needing to be discarded, whereas, by contrast in dysmorphometrics, the variables of interest are the atypical variations with the typical variations being the confounding variables. However, the challenge remains to define and identify atypical variation whilst compensating for the confounding typical variation.

#### Modelling form differences as outliers

A morphological abnormality can be seen as a difference in form that is inconsistent, discordant, and/or atypical. This definition inherently implies two important aspects. Firstly, they are only relatively defined and can only exist given prior knowledge of what is normal, consistent, harmonious, and/or typical. Hence, a representative norm is required. Secondly, the difference is required to be significantly distant from the norm. Employing this definition, the key point is thus to model form differences as outliers.

Most often the studied abnormalities are spatially localized and do not extend over the whole region of interest. In morphometrics such abnormalities will be represented by an ensemble of landmarks positioned 'suspiciously' compared to the norm, while the other landmarks are positioned as predicted by the norm. In order to distinguish the 'suspicious' or outlier landmarks from the 'normal' or inlier landmarks, during a superimposition, only the inlier landmarks need to be optimally aligned in the LSS sense and used to estimate the model metric if necessary. Hence, an appropriate robust superimposition and model metric estimation is required. Although many of the estimator choices mentioned in the previous section can be used to obtain such a robust placement of landmarks they, unfortunately, model outliers only implicitly. In other words, these superimpositions are robust against outliers, as in not influenced by them, but they do not allow for a meaningful outlier flagging/detection mechanism. Furthermore, they do not allow for a (robust) Procrustes model metric estimation as their underlying distributions typically deviate from Gaussian distributions. Instead, dysmorphometrics models outliers explicitly using outlier processes. A problem formulated in terms of explicit outlier-processes can be converted or viewed in terms of robust estimators [32]. An outlier process formulation, however, is more general than the original robust estimator. For example, due to the explicit nature of the outlier process, constraints on the spatial organization of the outliers can be formulated [33]. Based on the relationship with robust estimators defined in [32], equivalent M-estimators can be determined to analyze the influence of outliers on the superimposition.

#### Extending ML-estimators with outlier-processes

In practical applications, the assumed perturbation model in ML-estimators is only an approximation to reality, and estimation of the parameters should not be severely affected by the presence of unusual form differences. Indeed, the probability of an outlier according to the perturbation model is very low because the *outlier cannot be explained *by the model and therefore $\underset{Pr\left(d_{j}|\theta \right)->0}{\text{lim}}\text{log}Pr\left(d_{j}|\theta \right)=-\infty$. In order to deal with outliers a principled approach is adapted from [33]. The underlying idea is to model inliers and outliers as separate random variables. Inliers/Outliers are landmarks whose displacements have been generated by an inlier/outlier-process with associated inlier/outlier-distribution. The complete process, having generated all displacements, with associated complete distribution combines both inlier- and outlier-processes through the introduction of a latent variable *z_j _*that signals whether the residual error *d_j _*is an outlier (*z_j _*= 0) or inlier (*z_j _*= 1). This is achieved using mixture modelling and the result is an augmented superimposition in which landmarks are superimposed and outliers are flagged at the same time.

Given the observed landmarks, a joint estimation is now presented where besides the transformation parameters *θ *also the outlier map of latent variables or unobserved data *Z *= {*z_j_|j *= 1,..., *K*} is to be estimated. A popular tool for statistical estimation problems involving unobserved or incomplete data is the Expectation-Maximization (EM) algorithm ([34,35]). Herein, the likelihood of transformation parameters can be re-written in terms of the latent variables: $Pr\left(D|\theta \right)=\underset{Z}{\sum }Pr\left(D,\;Z|\theta \right)$. EM then produces a sequence of parameter updates $\left\{{\hat{\theta }}^{\left(t\right)}|t=0,1,\ldots \right\}$ by alternating two steps: the E-step and the M-Step. In the E-step, the latent variables are estimated using the conditional expectation resulting in the so-called Q-function. In the M-step, the Q-function is maximized generating a new update for the transformation parameters. Note that the Q-function is the equivalent robust M-estimator for the superimposition with an explicit outlier-process formulation.

The challenge is to formulate all likelihoods and distributions involved taking into account certain assumptions and prior knowledge of the augmented superimposition problem. In Appendix C we give such a detailed formulation and derive a practical extended Procrustes (Ext-P) ML-estimator. Here, we only summarize the practical Ext-P ML-estimator.

#### A practical extended Procrustes ML-estimator

As an example ML-estimator extension, we start from the Procrustes ML-estimator assuming a zero-mean Gaussian perturbation model with i.i.d landmark displacements (using the NLL in (5)). Hence, the model metric reflects additive white Gaussian noise (AWGN) with a single parameter *σ*. This constitutes the inlier-distribution *Pr_i_*(*x*). For the outlier-distribution several choices can be considered. If some knowledge about the associated distribution of the outlier generating process is given then this can be used. In most cases, however, this knowledge is not known and could be estimated as well [33,36]. However, we simply assume the outlier-distribution to be uniform such that *Pr_o_*(*x*) = *δ *with 0 <*δ *< 1. In this simplified case, the Q-function, written down as an M-estimator (7), has a *ρ*-function equal to:

(8) $$\rho \left(d_{j}\right)=\frac{b_{j}}{2\sigma^{2}}d_{j}^{2}+b_{j}\text{log}\sqrt{2\pi }\sigma -\left(1-b_{j}\right)\text{log}\delta$$

(9) $$b_{j}=E_{Pr\left(Z|D,{\hat{\theta }}^{\left(t\right)}\right)}\left[z_{j}\right]=\frac{Pr_{i}\left(d_{j}|{\hat{\theta }}^{\left(t\right)}\right)}{Pr_{i}\left(d_{j}|{\hat{\theta }}^{\left(t\right)}\right)+\lambda }$$

(10) $$\lambda =\frac{1}{\sqrt{2\pi }\sigma }\text{exp}\left(-\frac{1}{2}\kappa^{2}\right)$$

In (8) we see that for inlier landmarks (*b_j _*= 1) the Ext-P ML-estimator is equal to the original Procrustes ML-estimator (5). Furthermore, an outlier landmark (*b_j _*= 0) does not contribute to the estimation of the transformation parameters *θ *or model metric parameter *σ *because log *δ *is independent of both. From (9), we see that the estimation of the latent variables during superimposition is dependent on the estimated *σ *of the inlier-distribution and on a single parameter *λ *that codes for prior knowledge on the outlier distribution. Changing *λ *changes the amount of outliers versus inliers. A higher value suggests more outliers, while a lower value suggests more inliers. A proper choice of this parameter is therefore required, but can prove to be challenging. This choice is made easier and, more importantly, statistically relevant in (10) following [37].

**Figure 3:**
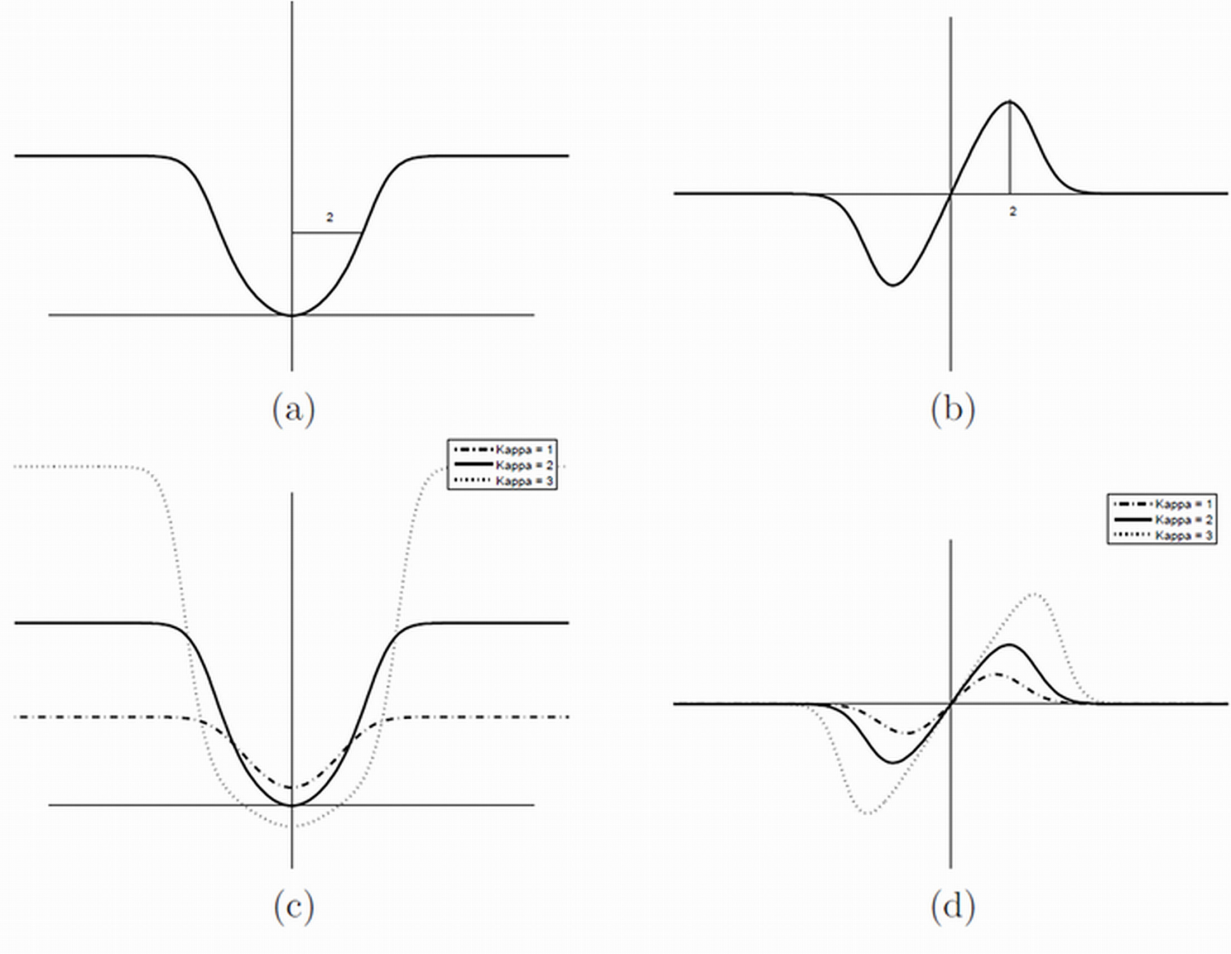
**Equivalent M-estimator**. Equivalent M-estimator and outlier influence functions for the augmented superimposition with AWGN modelling (a) and (b) with *σ *= 1 and *κ *= 2. (c) and (d) similarly but for *σ *= 1 and *κ *= 1, 2, 3 or equivalently *κ *= 2 and *σ *= 0.5, 1, 1.5 values, suggesting automatic adaptation of the M-estimator in function of the inlier-distribution or typical variation.

In statistics, an observation can be called atypical with respect to a given normal distribution if its (Mahalanobis) distance exceeds a predefined threshold. Therefore, in (10) *λ *is made inlier-distribution dependent and is re-parameterized using the more interpretable prior outlier parameter *κ*. The actual choice of *κ *is equivalent to the choice of a statistical significance level above which local form differences are considered atypical compared to the typical Gaussian (inlier-) distribution. For example *κ *= 2 and *κ *= 3 suggest a significance level of p = 0.05 and p = 0.001, respectively. Figure 3 depicts the *ρ*-function (8) (Figure 3(a)) and its influence function (Figure 3(b)). It is a continuous approximation of the truncated quadratic function (thus having better second derivate properties, which is important for reasons of optimization and convergence) and resembling the Tukey-biweight function. Note that the width of the *ρ*-function is equal to *κ × σ *and thus completely defined in terms of the inlier-distribution and prior outlier significance parameter. In Figure 3(c) and Figure 3(d) the *ρ*- and influence functions are depicted for different values of *κ *with fixed *σ *or, equivalently, different values of *σ *with fixed *κ*. It is observed that, apart from the intuitive choice of *κ*, a meaningful and adaptive outlier flagging mechanism is provided. Indeed, inlier landmarks, encoding for typical form differences, are fitted in the LLS sense as in the original Procrustes-fit and thus provide an estimate of typical form variation following the assumed perturbation model. Outlier landmarks are then identified in terms of being significantly atypical compared to the estimated typical variation. The resulting Ext-P ML-estimator with significant outlier detection is used throughout the results section with a fixed parameter setting *κ *= 2, reflecting a common choice for statistical significance (p = 0.05) in biology.

## Results

In this section, we focus on the biological relevance of the results only and additional computational comparisons such as time complexity can be found in Appendix D. Throughout the results, human faces are used as example biological forms of interest (Appendix A). They are the biological billboard of our identity, underlying genes, and environmental exposures. Our visual-cognition system is very adept and capable of discriminating between different faces and identifying or classifying facial abnormalities. Furthermore, facial abnormalities have been studied in depth in the literature enabling knowledge-based validation of our results.

### Dysmorphometrics for abrupt facial changes

**Figure 4:**
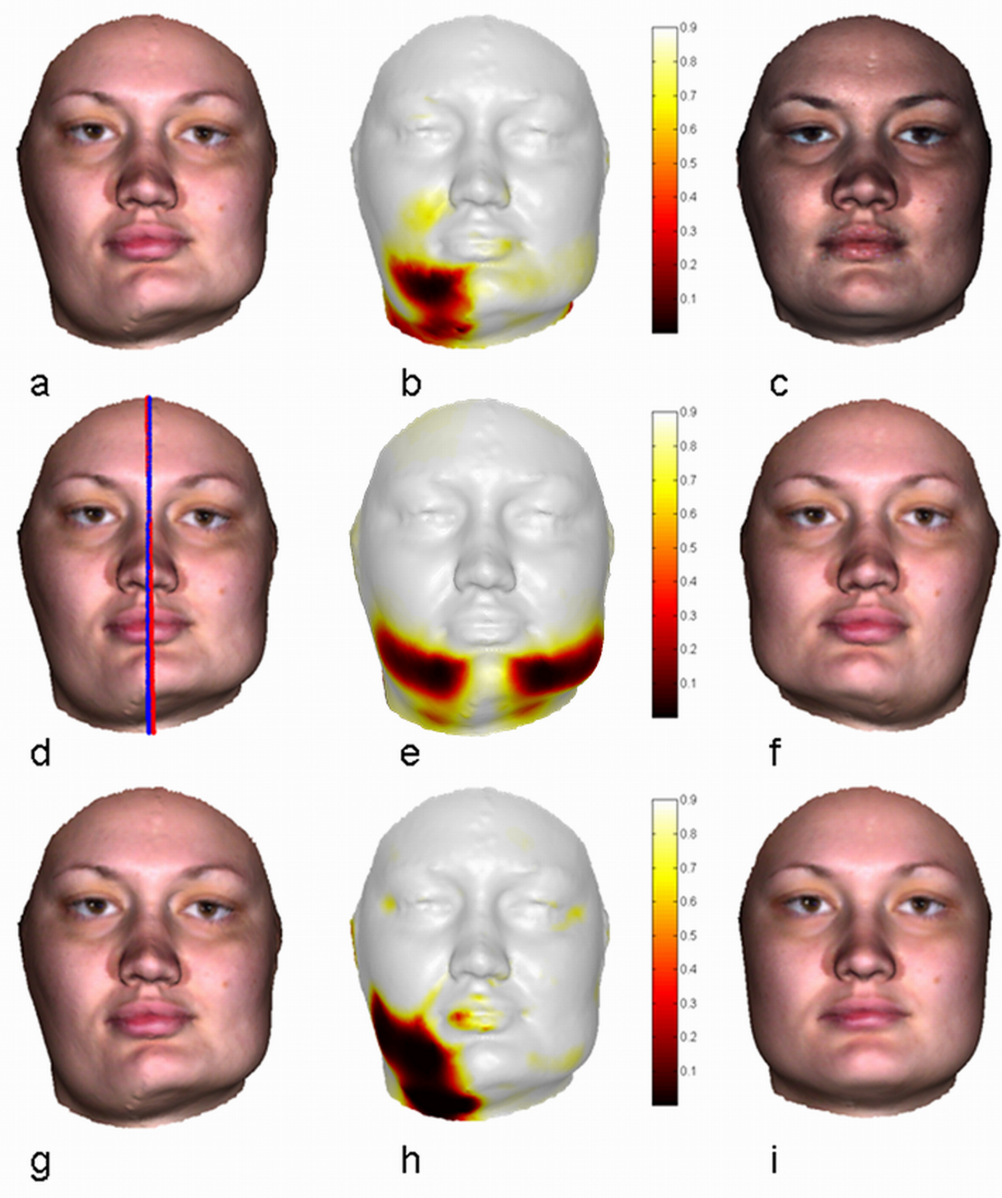
**Facial form change, asymmetry and discordancy**. Facial form change, asymmetry and discordancy of a 19 year old woman with a right hemimandibular hypertrophy. Top Row: Assessment of facial change due to surgical intervention. (a) Pre-surgical facial surface, representing the norm. (b) Dysmorphogram of facial change, depicting the features that changed. (c) Post-surgical facial surface. Middle Row: Assessment of facial asymmetry. (d) Original facial surface being the norm. With a robustly obtained mid-facial line (blue) and skewed symmetry line (red) obtained with an original Procrustes-fit. (e) Dysmorphogram of facial asymmetry, depicting the asymmetrical features. (f) Mirrored facial surface, according to the blue mid-facial line. Bottom Row: Assessment of facial discordancy (g) Pre-surgical facial surface to assess (i) Norm-equivalent of the Pre-surgical facial surface, being the norm (h) Dysmorphogram of facial discordancy, depicting the features that are considered abnormal compared to a normative reference population.

Abrupt changes in form may occur for many reasons and are highly variable making their measurement challenging. The Pinocchio effect is such a (synthetic) toy-example. Here, the true change was perfectly identified in the dysmorphogram (Figure 1(d)) using the proposed Ext-P ML-estimator. Biomedical examples of abrupt changes in facial form include deformations associated with surgical interventions or traumatic events. The capacity to measure the effects of surgical treatment over time has been long sought after to audit outcomes and evaluate relapse [23]. Such measurements provide feedback and insight allowing for reflection on surgical improvements. An example of a surgical intervention is illustrated in the top row of Figure 4. It shows a 19 year old woman treated for facial asymmetry coincident with right hemimandibular hypertrophy. The discrepancy in the lower mandibular border was corrected with an ostectomy and a wedged Le Fort1 osteotomy to resolve occlusal cant. 3D images were taken pre- (Figure 4(a)) and post-treatment (Figure 4(c)).

Visual comparison of the pre and post-surgery images clearly shows regions that have been altered by the intervention. In order for the superimposition to reflect true changes in form, it has to be performed based on the unaltered regions only. A dysmorphometric solution to this is to define the 'norm'; in this case, e.g., the pre-surgical situation. Then, an Ext-P ML-estimator is used for the superimposition of the post-treatment situation onto the norm. The idea is that outlier-landmarks are situated in regions that changed, while inlier-landmarks are located in the unchanged regions. The variation encoded in the inlier-landmarks should ideally be zero as in the Pinocchio example, but in reality this is never the case. This is because errors in the 3D scanning and quasi-landmark mapping procedures introduce noise or small perturbations on the landmark locations. Here, the level of this noise is considered typical variation (of no interest) around the norm configuration and is modeled by the AWGN inlier-distribution of the Ext-P ML-estimator. Outliers or atypical differences are then defined as significantly different with regard to the estimated noise-level. The resulting dysmorphogram is shown in Figure 4(b). It depicts a continuous-valued [0,1] spatial map of the local regions that are significantly different ('yes', non-white regions) or not ('no', white regions). According to the intervention that took place these accurately reflect the anticipated anatomical changes. Similar examples can be found in related work [23].

### Dysmorphometrics for facial asymmetry

Bilateral symmetry often occurs in organisms and is defined with respect to reflection across the midsagittal plane dividing a perfectly bilaterally symmetrical organism into equal right and left halves. During development in vertebrates imbalances in growth will inevitably result in some degree of asymmetry. Mild facial asymmetries are thus common in typical growth and development [38]. Severe and pathological asymmetries, on the other hand, are a feature of disordered growth as a consequence of genetic and/or environmental causes [39].

A protocol, grounded in geometric morphometrics, for the measurement of objects displaying bilateral symmetry consists in undertaking a LSS Procrustes-fit of landmark configurations and their mirror configurations [40,41]. However, when the asymmetry in the face increases locally and develops abnormally, as in the pre-surgical presentation of the case shown in Figure 4(d), the original Procrustes-fit becomes influenced by it. To obtain a robust assessment of asymmetry the superimposition involved has to be done based on the more symmetrical regions only. A dysmorphometric solution is to define the original configuration as the 'norm'. Then, an Ext-P ML-estimator is used for the superimposition of the mirror configuration onto the norm. The idea now is that outlier-landmarks are situated in regions that are asymmetrical, while inlier-landmarks are located in symmetrical regions. Again, errors in the 3D scanning and quasi-landmark mapping procedures introduce noise on the landmark locations modeled by the AWGN inlier-distribution.

The resulting dysmorphogram of the asymmetry case is shown in Figure 4(e). As a by-product, after the superimposition, a robust estimate of the midsagittal plane can also be obtained (blue line in Figure 4(d)). This 'extended' asymmetry assessment protocol has been used in related work [22,42]. There, the goal was to detect disordered facial growth patterns in individuals characterized by asymmetries with reference to the individual asymmetry variation found in the general population rather than to some ideal of perfect symmetry, which rarely exists.

### Dysmorphometrics for facial discordancy

Given a population of interest, an individual is concordant with that population if it is within the boundaries of variation of the population. Stated differently, a concordant form is in harmony with the population. Form discordancy, on the other hand, is the lack of harmony. Biologically, this is manifested as the type of facial abnormalities that are best known in craniofacial disorders and dysmorphologies. Assessment of such disorders affecting facial morphology is typically performed compared to 'normality'. This, however, presents two major challenges. The first is to define normality or harmony in facial form. The second is to define the dysmorphic face with respect to normality by identifying and localizing the discordancy in the form of the face.

A morphometric approach to this problem is to establish both normal and abnormal population databases [43] and to assign a given individual to the most plausible population, conceptually similar to a taxonomic discrimination in systematic biology. Normal variation can be learned starting from a proper reference dataset consisting of healthy individuals without pathology. The collection of abnormal population data however often proves to be more difficult and impractical particularly when dealing with rare and highly variable situations. A dysmorphometric formulated solution approaches the problem without the need to compile databases representing abnormal populations as follows: Firstly, the normal population is chosen as the 'norm'. Secondly, an Ext-P ML-estimator is used for the superimposition of a configuration under investigation onto the norm. Here, the 'population norm' is represented as a Point Distribution Model (PDM) [10] using a principal component analysis (PCA) around the consensus configuration of the population (Appendix B). One of the advantages of using a PDM is that the within-population variation becomes part of the transformation model used during superimposition. As such, typical variation in the population is treated and compensated for in the exact same way as other confounding variables of no interest like orientation and position. The result of this superimposition is the creation of a norm-equivalent configuration, which is the harmonious counterpart of a given form configuration and can be considered as an individual-specific typical or normalized reference. Stated differently, the consensus configuration of the population is allowed to change within the boundaries of typical variation to reflect the given configuration as much as possible.

The superimposition or the creation of the norm-equivalent of a given dysmorphic face has to be performed on regions in the face that are in harmony with the normal population. From a dysmorphometric point of view, the idea is that outlier-landmarks are situated in regions that are discordant, while inlier-landmarks are located in concordant regions. Again, errors in the 3D scanning and quasi-landmark mapping procedures introduce noise or small perturbations on the landmarks locations. The level of this noise is considered typical variation (different to the population variation) and as before is modeled by the AWGN inlier-distribution. Outliers or atypical differences are then defined as significantly different w.r.t. the norm-equivalent configuration based on the estimated noise-level, therefore reflecting significant discordancy after compensating for within-population variation. Note that by using the PDM within the transformation model of the superimposition, the simple but practical Ext-P ML-estimator only modelling AWGN can be used to separate landmarks into concordant and discordant.

**Figure 5:**
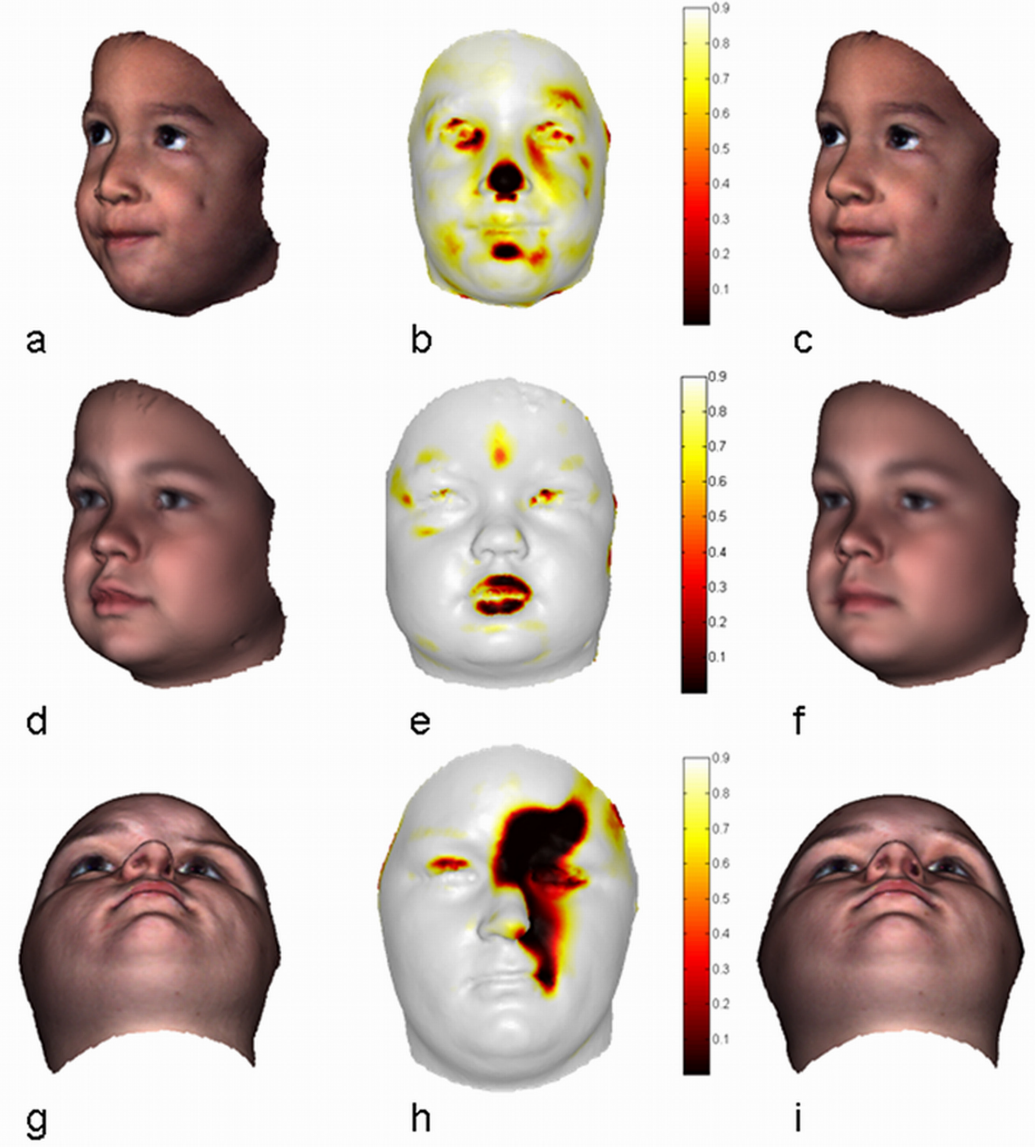
**Facial discordancy of craniofacial disorders & syndromes**. From left to right: original facial surface, dysmorphogram of facial discordancy and norm-equivalent. From top to bottom: assessment of persons with mild Treacher-Collins syndrome, a Lysosomal Storage Disorder and a Parry-Romberg syndrome. A general note: eyes and areas in the face covered by hair, e.g. eyebrows, always contain 3D surface mesh-artifacts (due to limitations of current scanning technology) such that spurious outliers are visible in these areas. These are to be interpreted with caution.

For the lying Pinocchio toy-example (Figure 1(b)) the norm-equivalent according to a clinically normal population turns out to be the honest Pinocchio (Figure 1(a)) (which was the consensus of that population) with a dysmorphogram (Figure 1(d)) correctly identifying the nose as significantly discordant. Assessment of facial discordancy is further demonstrated in four different cases shown in Figure 4 (bottom row) and Figure 5. From left to right, the facial configuration under study, the dysmorphogram and the norm-equivalent configuration are displayed. The norm-equivalent of each case is clearly a patient-specific norm, which has not been previously available and is proving to be of value in clinical practice for treatment planning and auditing purposes. The first case (Figure 4(g,h,i)) concerns a 19 year old woman with right hemimandibular hypertrophy, treated for facial asymmetry. The dysmorphogram, as expected, localizes the highly asymmetric right lower mandibular as discordant but indicates a displacement as well of the mandible to the contralateral side and alveolar compensation in the maxilla on the affected side. The second case (Figure 5(a,b,c) is a child with a (mild) Treacher-Collins Syndrome (TCS), a rare genetic disorder characterized by craniofacial deformities with occurrence prevalence of 1:10,000 [44]. The dysmorphogram revealed regions known to be affected in TCS including malar, zygomatic, and periorbital regions. In addition, the nasal tip was highlighted, which has previously been described but not considered a major symptomatic feature (GeneTests, http://www.genetests.org). The third case (Figure 5(d,e,f)) is a person suffering from Lysosomal Storage Disease (LSD, MPSII), which is a rare inherited metabolic disorder that results in accumulations in glycosamminoglycans (GAGs) with a prevalence of <1:100,000 [45]. People with this condition have been described as having 'coarse' facial features; the dysmorphogram demonstrated the fullness to the lips for example caused by accumulated GAGs. The last case (Figure 5(g,h,i)) is a person with a Parry-Romberg Syndrome. This is a rare hemifacial atrophy disorder characterized by progressive degeneration of the subcutaneous tissues and fat that can also involve bone, cartilage and muscle. This progressive condition often affects the left maxillae adjacent to the nose progressing to the corner of the mouth, around the eyes and brow but may vary from case to case as it appears to occur randomly with unknown etiology [46]. The dysmorphogram illustrates the extent of the disease in this young woman, in whom the affected area appears to be confined to distinct connective tissue septa in the face. All these cases illustrate features that have been previously known as distinctive features of the condition. However, new and highly relevant spatial information can be identified and quantified as well. This transforms previously descriptive dysmorphology to much more informative quantitative dysmorphometrics.

## Discussion

The study of form using quantitative, morphometric descriptors is fundamental to many biological studies. Emerging technologies of 3-dimensional (3D) scanning and geometric morphometrics are providing the means to establish objective criteria which can be used for phenotypic investigations in combination with epigenetic investigations [47,48]. Here, we have introduced dysmorphometrics as a means to identify and quantify unusual form differences like abnormalities that are typically encountered in biomedical sciences. Dysmorphometrics builds upon existing geometric morphometric techniques, but models form differences explicitly as outliers. Applying a test for outliers is referred to as a test of discordancy [49] and the practical Ext-P ML-estimator clearly resembles known statistical tests for outliers. For example, the same result could have been obtained by applying a Grubbs' test using the inlier-distribution without the need to explicitly define an outlier-process and associated distribution. However, the explicit case is more general and open to further assumptions or prior knowledge about the outliers. The potential of this is strong and is still to be explored. The key is to model the patterns and their analysis as biologically relevant based on the right assumptions.

The requirement for robustness during the analysis of unusual form variation has been acknowledged by many others. For example, [50] advised the need for caution to be taken into account when comparing preoperative with postoperative asymmetry scores in faces. This was because the achievement of 'best-fit', using the LLS Procrustes superimposition, was influenced by severe asymmetries in the preoperative situations. This resulted in spurious changes and unrealistic reduction of the asymmetry postoperatively in regions not affected by the surgical intervention. A popular way to address this problem in practice is to work with "stable" landmarks only, based upon which the superimposition is then performed. For example, the superimposition is done on predefined areas in the face like the nose ridge in [51] or by carefully indicated landmarks as in [39] and in [52]. Another strategy is to perform a LSS superimposition first, then remove parts according to a threshold and redo the superimposition using the remaining parts [53]. All these approaches try to achieve the same result as a robust superimposition. However, they introduce subjectivity by the obligation to manually select a region, landmarks or threshold of interest. They are heuristic and different for different superimposition tasks. Unfortunately, at the time, the known repeated median method that was used for resistant fitting [12] lacked proper mathematical underpinning and convergence behavior compared to their original LSS solution and was therefore never advocated as an alternative. In contrast, the proposed modelling methodology of dysmorphometrics results in an adaptive and robust superimposition with meaningful outlier detection. The underlying mathematical model is an extension of the original theory of Procrustes ML-Estimators [27]. Furthermore, it is shown that behavior against outliers equates that of re-descending M-estimators, which are a popular and efficient class of estimators in robust statistics. However, the difference here is that the underlying Gaussian perturbation model assumption is kept intact. Alternatively, this is also achieved using the previously mentioned techniques of heteroscedastic variance estimation during superimposition [26,31]. However, these techniques by themselves do not allow for a robust model metric estimation, as the metric is stretched to include any higher variances caused by outliers. This inclusion does not enable separating outliers from inliers and so might be undesirable for further analysis. An interesting extension, however, would be to combine both the techniques of heteroscedastic variance estimation and dysmorphometric outlier flagging.

In the assessment of discordancy, which inspired the development of the concept of dysmorphometrics, contrasts in strategy with morphometric analysis are apparent. For example, [43] and [54] build distributions of form for both typical and atypical populations. To assess an individual a closed-classification is then performed. However, the individual is always attributed to either one of the given populations, even in the case when it does not belong to any of them. Furthermore, this morphometric approach loses the power to individualize and can only visualize population differences expressed as a difference between averages or a net difference. In contrast, a dysmorphometric approach enables an open-classification, which is less restrictive and has a greater range of applications. It also allows for an individual-specific assessment and visualization of a problem or hypothesis. To conclude, the essence of dysmorphometrics is the ability to identify and measure the unknown abnormality, if any; with a norm reflecting what is known. This enables an alternative research strategy. Whereas morphometrics is typically used in a deductive research approach, where a general theory is formed and typical data observations are collected to test hypotheses, dysmorphometrics, on the other hand, provides for an exploratory research approach. An initially unknown, quantified observation is then made that can be subject to further testing by directed data collection from which a general theory is derived. A good example is the extent of the Parry-Romberg disease appearing to be confined by known connective tissue septa in the face. Hypotheses to explain this single observation are to be validated by collecting similar case data.

The use of dysmorphometrics is limited by two constraints. Firstly, but most importantly, an appropriate norm must be established. Hence, a straightforward comparison between two different individuals without taking population variation into account, e.g., cannot be done because neither one of the two individuals can be chosen as the norm. Additionally, in case of a population-based norm, it may prove challenging to decide if an individual belongs to the norm or not and will be entirely dependent on the application. Secondly, the number and extent of form abnormalities cannot exceed the breakdown point of the outlier detection scheme employed. The breakdown point of a detection scheme is the percentage of data allowed to be outliers before they become undetectable.

## Conclusions

Morphological abnormalities are often encountered and of great interest in biomedical sciences and include, e.g., deformations in form due to congenital malformation and/or environmental constraints as well as large changes in form associated with traumatic injuries and surgical interventions. In current morphometrics, the means to identify and quantify such unusual form differences remains limited. To address this shortcoming, dysmorphometrics was introduced, which is a novel and unexplored concept that augments current geometric morphometrics to deal with, quantify and spatially map form abnormalities to facilitate their analysis. Essentially, an abnormality implies the existence of a data pattern that cannot be explained by typical patterns. As such, dysmorphometrics models form differences explicitly using outlier processes, resulting in adaptive and robust superimpositions. Furthermore, dysmorphometrics builds upon existing techniques such that the underlying mathematical model is a straightforward extension of the original theory of Procrustes ML-Estimators.

Throughout the results, outliers were defined above an estimated noise-level, as modeled in a practical Ext-P ML-estimator, therefore reflecting significant differences in form with a biological meaning. The results are unique and illustrate the power of this technique to reveal unusual form differences given only normative data either representing a single individual or a population. In the case of a population norm, variation within the population is considered of no interest and is treated as a confounding variable just like orientation and position differences. Dysmorphometrics can generate an individualized quantification of form abnormalities and leads to alternative and more informative population comparisons and research strategies. The analysis of multiple dysmorphograms for example can lead to novel and unexplored statistics in morphometric analysis.

## Appendix A: Facial Data Acquisition

Human faces are used as the biological data of interest for which appropriate Ethics approval was received: (1) *The Characterization of 3-Dimensional Facial Profile in Young Adult Western Australians *was granted from the Princess Margaret Hospital for Children (PMH) ethics committee (PMHEC 1443/EP) in Perth, WA, Australia. (2) *Establishment of Identity from Quantitative Analysis of Facial Characteristics (Digital 3D facial modelling) *was granted from the University of Melbourne, human research ethics committee (HESC 050550.1) in Melbourne, VIC, Australia.

3D facial images of 800 healthy young people as well as patients between the ages of 5-25 were collected using a 3dMD facial scanning system. An anthropometric mask (AM) [22,23] was mapped onto the 3D facial images in a (quasi) anatomical manner using a non-rigid surface registration algorithm based on implicit functions described and validated in [25]. As a result all the points defined in the AM were automatically indicated on each facial surface separately in a consistent way. Consequently, spatially-dense (~10.000) quasi-landmarks are known on all 3D facial images.

## Appendix B: Transformation Model

The transformation model stipulates how landmark configurations can be placed onto each other and reflects prior knowledge on the superimposition problem. Sometimes, not all transformations are feasible or realistic so that certain fitting constraints can be imposed to ensure that the superimposition behaves according to the prior knowledge. These constraints, inducing transformation regularizations, can be modeled probabilistically using a Gibbs prior distribution [10] on the parameters *θ*, restricting the space of possible solutions:

(11) $$Pr\left(\theta \right)=\frac{1}{Z}e^{-||L\left(\theta \right)|{|}^{2}}$$

This distribution expresses the probability of a certain transformation parameter setting *θ*, within the range of possible parameters, favoring more plausible settings. *Z *is a normalization constant and *L *is an operator defined on the space of parameter values representing the regularization as a squared norm. Inclusion of the prior model into the likelihood formulated superimposition follows a Bayesian inference strategy and results in multiplying *Pr *(*D|θ*) by (11). After taking the negative log likelihood, an extra term ||*L*(*θ*)||^2 ^is therefore added to equations (2-6).

### Single norm transformation model

The simplest transformation model is the class of rigid transformations, only compensating for overall differences in pose by global translation, rotation and, if shape instead of form comparison is wanted, also scaling:

(12) $$T\left(\overset{`}{C},\theta \right)=sR\overset{`}{C}+t$$

*R *is a rotation matrix parameterized using three Euler angles, *t *is a 3D translation vector and *s *is a scaling factor. This transformation model is well-known and extensively used in geometric morphometrics. It is also used in dysmorphometrics when dealing with a norm that is defined based on a single configuration as in the scenarios of time related form changes and asymmetry assessments of individuals. Generally, no particular rotation, translation and/or scaling is favored, hence due to the rigidity of the transformation model the regularization simply reduces to ||*L*(*θ*)||^2 ^= 0, such that (11) is a constant term (and therefore omitted) not influencing the ML-estimation of the parameters *θ*.

### Population norm transformation model

In the case of a population defining the norm, the same rigid transformation model (12) can still be used. In this scenario, a Generalized Procrustes-fit of all the configurations in the population is performed and the norm consists of the resulting consensus configuration in combination with the full covariance matrix Ω around it. The covariance matrix is the model-metric coding for typical shape variation amongst individuals in the normal population and can be plugged straight into a Procrustes ML-estimator without the need to make any of the simplifying assumptions to create equations (2-6). Doing so, measures the Procrustes distance between the consensus configuration and a given configuration relative to the within population variation known as the Mahalanobis distance in multivariate statistics as stated in [55] whilst referring to [56]. The advantage is that the model metric is given based on a population sample and does not need to be updated or estimated during superimposition. Furthermore, the analysis is a clear multivariate analysis taking into account the complete configuration and within population variation to determine harmony or lack thereof. The disadvantage, however, is that it becomes difficult to localize the discordant regions in the configuration and one is limited to keeping or rejecting the complete configuration as a whole. Furthermore, the Mahalanobis distance requires an inversion of the full covariance matrix that becomes computationally expensive and even practically impossible when dealing with a vast amount of landmarks.

An alternative for a population norm is the use of a Point Distribution Model (PDM) [10]. A PDM is a model for representing the mean geometry of a configuration and some statistical modes of geometric variation inferred from a training set of configurations. First a Generalized Procrustes-fit of all the configurations in the population is performed as before. The consensus configuration represents the mean geometry. Subsequently, a principal component analysis is done on the aligned data generating a ranked set of principal components representing the modes of geometric variation [57]. Doing so, allows for new configurations to be constructed based on the geometric mean and a linear combination of modes of geometric variation:

(13) $$\overset{`}{C}=\bar{C}+\overset{D}{\underset{k=1}{\sum }}U_{k}c_{k}$$

$\bar{C}$ is the consensus configuration, *U_k _*is the k^th ^eigenvector or principal component of the covariance matrix Ω, *c_k _*is the k^th ^PDM parameter and reflects the loading or contribution of the k^th ^principal component and *D *is the number of principal components used in the PDM. It is quite common to use only the top part of the principal components explaining, e.g., 98% of the total variance in the population (under the assumption that the last 2% corresponds to biologically insignificant variance due to random errors or artifacts). Finally, plugging (13) into (12) defines the PDM based transformation model $T\left(\bar{C},\theta \right)$ for a population norm. It starts from the consensus configuration $\bar{C}$ and allows compensating for rotation, translation, scaling and typical variation differences as confounding variables to determine true differences in form, defined as outliers, with a given configuration *C*. Note that $\overset{`}{C}$ is the norm-equivalent configuration of *C *after ML-estimation. The full set of transformation model parameters *θ *to estimate are three Euler angles, three translation directions, a scaling factor and *D *PDM parameters. Without favoring any rotations, translations or scaling the transformation model regularization is completely dependent on the PDM parameters only and the eigenvalues *α *of the eigenvectors and can be defined as:

(14) $$||L\left(\theta \right)|{|}^{2}=\frac{1}{2}\overset{D}{\underset{k=1}{\sum }}\frac{c_{k}^{2}}{\alpha_{k}^{2}}$$

This is the Mahalanobis distance of a PDM parameter setting favoring more plausible configurations within the population sample and statistics. Stated differently, it restricts the PDM from diverging too far away from the consensus configuration taking into account the within-population variation.

It is interesting to note that when using a PDM the covariance matrix of the within-population variation is moved from being used as the superimposition metric to being part of the superimposition transformation model. This allows for an additional superimposition metric to be defined like the modelling of i.i.d AWGN in the practical extended Procrustes ML-estimator. Doing so, results in an analysis that remains multivariate in nature whilst being able to determine local regions of discordancy. They are then defined as being significantly bigger than the estimated noise-level after compensation for within-population variation in the norm-equivalent besides the other confounding variables.

## Appendix C: Augmented Superimposition

### Likelihood formulations

Given the observed landmarks a joint estimation is presented where besides the transformation parameters *θ *also the outlier map of latent variables or unobserved data *Z *= {*z_j_|j *= 1,..., *K*} is to be estimated. Following an Expectation-Maximization (EM) based estimation strategy, the likelihood of transformation parameters can be re-written in terms of the latent variables as:

(15) $$Pr\left(D|\theta \right)=\underset{z}{\sum }\text{Pr}\left(D,\;Z|\theta \right)$$

EM produces a sequence of parameter updates $\left\{{\hat{\theta }}^{\left(t\right)}|t=0,\;1,\ldots \right\}$ by alternating two steps: the E-step and the M-Step. In the E-step, the latent variables are estimated given the observed landmarks and the current update of the transformation model parameters. This is achieved using the conditional expectation generating the so-called Q-function:

(16) $$\begin{array}{llll} Q^{\left(t+1\right)} & =\underset{Z}{\sum }Pr\left(Z|D,{\hat{\theta }}^{\left(t\right)}\right)\text{log}\text{Pr}\left(D,\;Z|\theta \right)=\; & & \;\\ & E_{Pr\left(Z|D,{\hat{\theta }}^{\left(t\right)}\right)}\left[\text{log}\text{Pr}\left(D,\;Z|\theta \right)\right]\; & & \;\\ & \; & \end{array}$$

In the M-step, the Q-function is maximized generating a new update for the transformation parameters:

(17) $${\hat{\theta }}^{\left(t+1\right)}=argmax_{\theta }Q^{\left(t+1\right)}$$

If the latent variable is modeled as a stochastic variable the outlier map *Z *becomes a random map with an associated prior-distribution Pr(*Z*). Under the assumption that the outlier map is independent from the transformation model parameters:

(18) Pr(D,Z|θ)=Pr(D|Z,θ)Pr(Z)

*Focusing on the second factor in (18): *Let *P *be the prior probability of having an inlier (for example the fraction of form differences thought to be generated by the inlier-process) and let 1 *- P *be the prior probability of having an outlier. Then, assuming *Z *to be i.i.d we can specify *Pr *(*Z*) as a product of i.i.d. Bernoulli distributed variables *z_j _*:

(19) $$Pr\left(Z\right)=\overset{K}{\underset{j=1}{\prod }}P^{z_{j}}{\left(1-P\right)}^{\left(1-z_{j}\right)}$$

Note that extra constraints on the outliers can be incorporated here by choosing an alternative prior distribution *Pr*(*Z*). For example, in [33] spatially coherent outliers are modeled by considering a binary Markov-Random-Field with associated Gibbs prior.

*Focusing on the first factor in (18): *The complete likelihood under the assumption of independent landmarks can be specified by conditioning individual residual likelihoods on the state of the binary-valued latent variable *z_j _*signaling whether a local form difference was generated by the inlier-distribution *Pr_i _*(.) or the outlier-distribution *Pr_o_*(.):

(20) $$\text{Pr}\left(D|Z,\;\theta \right)=\overset{K}{\underset{j=1}{\prod }}Pr\left(d_{j}|z_{j},\;\theta \right)$$

with

(21) $$Pr\left(d_{j}|z_{j},\;\theta \right)=\left\{\begin{array}{cc} Pr_{i}\left(d_{j}|\theta \right), & if\;z_{j}=1\\ Pr_{o}\left(d_{j}\right), & if\;z_{j}=0 \end{array}\right)$$

or, equivalently using a mixture model notation:

(22) $$Pr\left(d_{j}|z_{j},\;\theta \right)=Pr_{i}{\left(d_{j}|\theta \right)}^{z_{j}}Pr_{o}{\left(d_{j}\right)}^{\left(1-z_{j}\right)}$$

The extension of a negative log-likelihood assuming a perturbation model only into a complete negative log-likelihood assuming an outlier-process as well is obtained by taking the negative logarithm of (18) using (19-22):

(23) $$\begin{array}{c} l\left(\theta \right)=-\overset{K}{\underset{j=1}{\sum }}z_{j}\left(\text{log}Pr_{i}\left(d_{j}|\theta \right)+P\right)+\\ \overset{K}{\underset{j=1}{\sum }}\left(1-z_{j}\right)\left(\text{log}Pr_{o}\left(d_{j}|\theta \right)+1-P\right) \end{array}$$

In the E-step the Q-function of (23) is obtained by dropping any constant terms independent from the parameters and by replacing the values of *z_j _*with their expected conditioned values:

(24) $$\begin{array}{c} E_{Pr\left(Z|D,{\hat{\theta }}^{\left(t\right)}\right)}\left[z_{j}\right]=\underset{z_{j}\in \left\{0,1\right\}}{\sum }z_{j}Pr\left(z_{j}|d_{j},{\hat{\theta }}^{\left(t\right)}\right)=\\ \text{Pr}\left(z_{j}=1|d_{j},{\hat{\theta }}^{\left(t\right)}\right)=b_{j} \end{array}$$

Using the Bayes Rule we can write:

(25) $$b_{j}=\frac{Pr\left(d_{j}|z_{j}=1,{\hat{\theta }}^{\left(t\right)}\right)Pr\left(z_{j}=1\right)}{\underset{x\in \left\{0,1\right\}}{\sum }Pr\left(d_{j}|z_{j}=x,{\hat{\theta }}^{\left(t\right)}\right)Pr\left(z_{j}=x\right)}$$

Based on (19) and (22) we then obtain:

(26) $$b_{j}=\frac{Pr_{i}\left(d_{j}|{\hat{\theta }}^{\left(t\right)}\right)P}{Pr_{i}\left(d_{j}|{\hat{\theta }}^{\left(t\right)}\right)P+Pr_{o}\left(d_{j}|{\hat{\theta }}^{\left(t\right)}\right)\left(1-P\right)}$$

### A practical extended Procrustes ML-estimator

As an example ML-estimator extension, we start from the Procrustes ML-estimator assuming a zero-mean Gaussian perturbation model with i.i.d landmark displacements (using the negative log-likelihood in (19)). Hence, the model metric reflects additive white Gaussian noise (AWGN) with a single noise parameter *σ*. This constitutes the inlier-distribution *Pr_i_*(*x*). For the outlier-distribution *Pr_o_*(*x*) several choices can be considered. If some knowledge about the associated distribution of the outlier generating process is given, than this can be used. In most cases, however, this knowledge is not known and could be estimated as well [33,36]. However, we simply assume the outlier-distribution to be uniform such that:

(27) $$Pr_{o}\left(x\right)=\delta \;\text{with}\;0<\delta <1$$

In this simplified case, the negative Q-function, written down from an M-estimator point of view, has a *ρ*-function equal to:

(28) $$\rho \left(d_{j}\right)=\frac{b_{j}}{2\sigma^{2}}d_{j}^{2}+b_{j}\text{log}\sqrt{2\pi }\sigma -\left(1-b_{j}\right)\text{log}\delta$$

with

(29) $$b_{j}=\frac{Pr_{i}\left(d_{j}|{\hat{\theta }}^{\left(t\right)}\right)P}{Pr_{i}\left(d_{j}|{\hat{\theta }}^{\left(t\right)}\right)P+\delta \left(1-P\right)}$$

From (28) we see that for inlier landmarks (*b_j _*= 1) the extended Procrustes ML-estimator is equal to the original Procrustes ML-estimator. Furthermore, an outlier landmark (*b_j _*= 0) does not contribute to the estimation of the transformation parameters *θ *or model metric parameter *σ *because log *δ *is independent of both. From (29), we see that the estimation *σ *of the latent variables during superimposition is dependent on the estimated of the inlier-distribution and also on the prior outlier distribution parameters *P *and *δ*. In order to simplify the choice of prior values, we combine these inter-dependent parameters into a single parameter *λ *= *δ*(1 *- P*)/*P *such that:

(30) $$b_{j}=\frac{Pr_{i}\left(d_{j}|{\hat{\theta }}^{\left(t\right)}\right)}{Pr_{i}\left(d_{j}|{\hat{\theta }}^{\left(t\right)}\right)+\lambda }$$

Furthermore, log *δ *can be substituted with log *λ *in (28) without any effect to the parameter estimation. Changing the parameter *λ *changes the amount of outliers versus inliers. A proper choice or fine-tuning of this parameter is therefore required, but can prove to be challenging. This choice is made easier and, more importantly, statistically relevant following [37]. Equation (30) reflects the posterior probability of a local residual to belong to the inlier-distribution, called the inlier-belief. The outlier-belief can then be defined similarly as:

(31) $$1-b_{j}=\frac{\lambda }{Pr_{i}\left(d_{j}|{\hat{\theta }}^{\left(t\right)}\right)+\lambda }$$

The outlier-belief exceeds the inlier-belief if *b_j _<*0.5 or $Pr_{i}\left(d_{j}|{\hat{\theta }}^{\left(t\right)}\right)<\lambda$ which is equivalent to $MD_{j}^{2}>-2\text{log}\lambda \sqrt{2\pi }\sigma \;\text{with}\;MD_{j}=d_{j}/)\sigma$the Mahalanobis distance. In statistics, an observation is called abnormal or atypical with respect to a given normal distribution if its (Mahalanobis) distance exceeds a predefined threshold. Because of its dependence on *σ *in, the Mahalanobis distance threshold above which a local form difference is considered abnormal changes when *σ *changes or is updated. Furthermore, observations are more easily rejected from classes with a broad distribution (e.g. overall big landmark displacements) than from classes with a narrow one, making the choice of *λ *dependent on the current superimposition problem, leading to a different *λ *setting for every different configuration to superimpose. Because of these problems it is not clear how *λ *should be chosen. Ideally, a spatial displacement should be considered abnormal if *MD_j _> κ*, where *κ >*0 is an explicit Mahalanobis distance threshold that is equal for all normal inlier-distributions alike. Therefore, taking into account the dependence on *σ*, *λ *is replaced by:

(32) $$\lambda =\frac{1}{\sqrt{2\pi }\sigma }\text{exp}\left(-\frac{1}{2}\kappa^{2}\right)$$

*λ *is now inlier-distribution dependent and re-parameterized using a more interpretable prior outlier-parameter *κ*. The actual choice of *κ *is equivalent to the choice of a statistical significance level above which local form differences are considered atypical (outlier) compared to a typical Gaussian (inlier-) distribution.

## Appendix D: Computational results

In this section the results obtained using an outlier process as formulated in the Ext-P ML-estimator are numerically compared to two commonly used alternative estimators: the popular Procrustes least sum of squares (LLS) and the resistant-fit or repeated median estimator.

**Figure 6:**
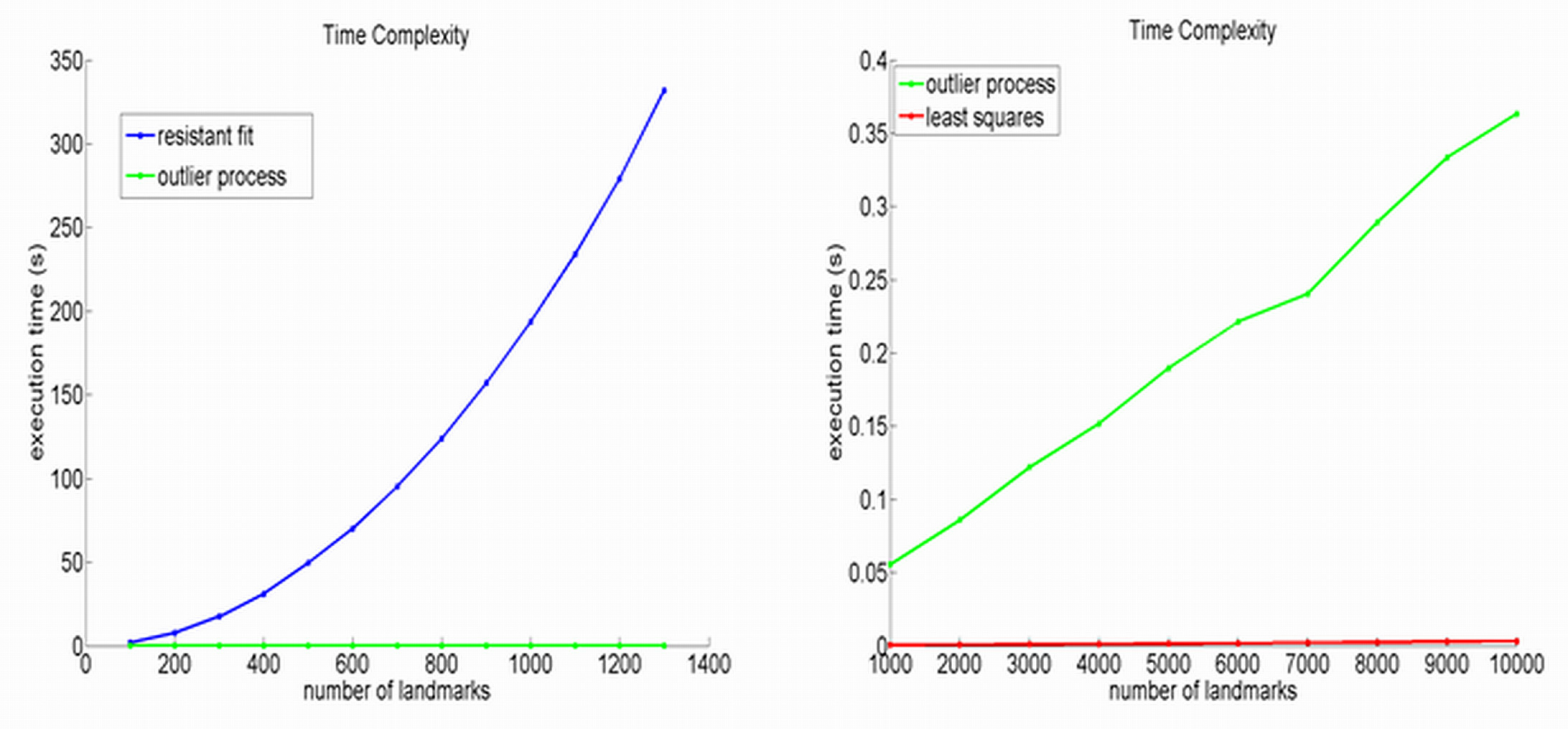
**Time Complexity**. Time complexity analysis of the Ext-P ML-estimator in function of the amount of landmarks against the resistant-fit (left) and Least Sum of Squares Procrustes ML-estimator (right).

Using increasing amounts of landmarks from the surgical intervention superimposition problem depicted in Figure 4(a) and 4(c), a time-complexity analysis is given in Figure 6. It is obvious that the resistant-fit suffers from an exponential time-complexity, whereas the LLS as well as the Ext-P ML estimator remain computationally practical even up to 10.000 landmarks.

**Figure 7:**
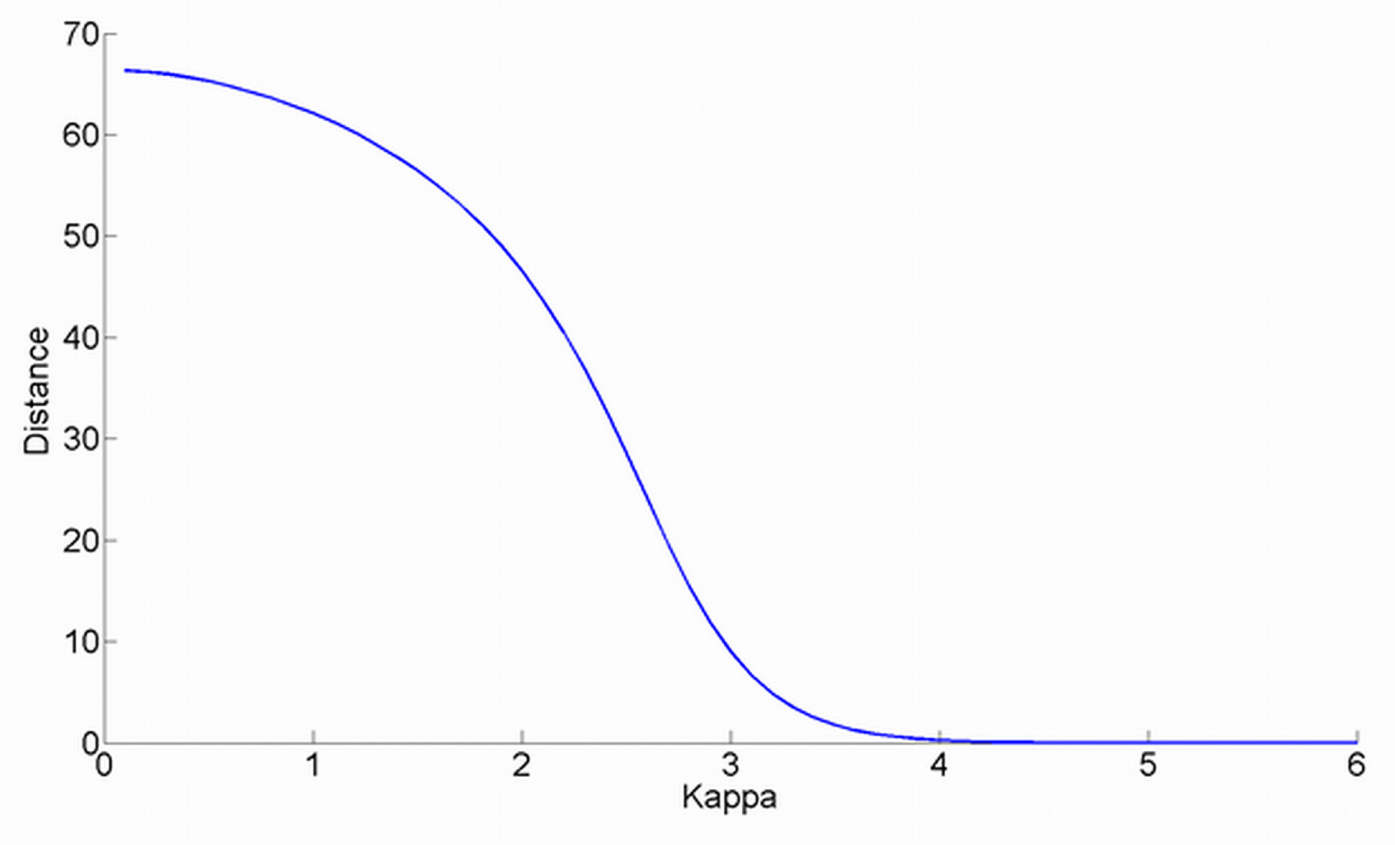
**Superimposition differences**. The difference, expressed as an Euclidean Distance between resulting landmark configurations, between the Ext-P ML-estimator solutions for varying values of *κ *and the Least Sum of Squares Procrustes ML-estimator solution, using the surgical intervention superimposition of Figure 4 (a-c).

Using the same surgical intervention superimposition problem, the Euclidean distance between landmark configurations after superimposition as a function of *κ *(Kappa) are given in Figure 7. For lower values of *κ*, the Ext-P solution differs from the LSS solution. A clear bending point is observed for *κ *in-between 2 to 3, corresponding to the typically used p-values of 0.05 and 0.001 for significance assessment. As expected, for higher values of *κ*, the Ext-P solution equates the LSS solution.

**Figure 8:**
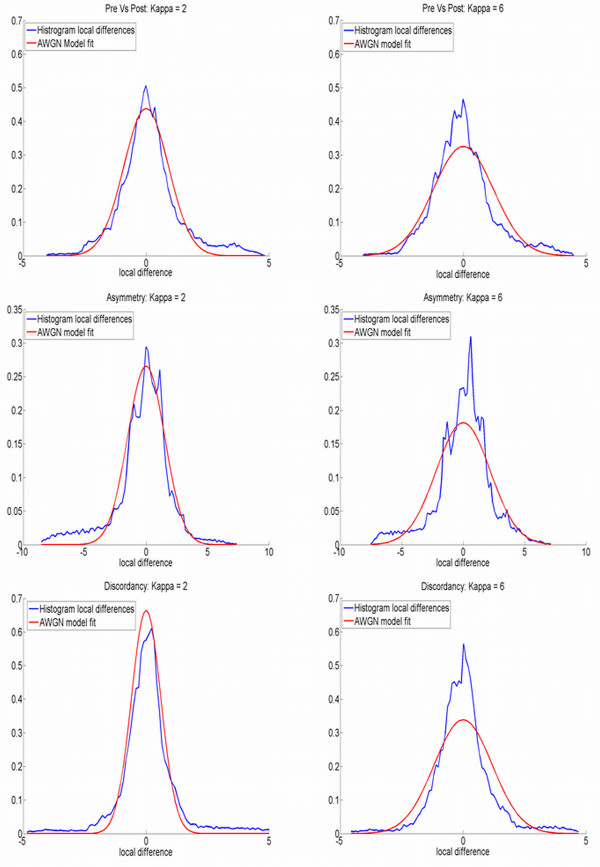
**Distribution of local form differences and overlaying estimated model**. Resulting distributions and overlaying AWGN model of the practical Ext-P ML-estimator for local form differences found on the three types of facial assessment for the subject illustrated in Figure 4. Top to Bottom: abrupt change due to surgical intervention, asymmetry and discordancy. Left Collumn: robust superimposition results using *κ *= 2. Right Collumn: unweighted superimposition results using *κ *= 6 (mimicking the LSS Procrustes ML-estimator as demonstrated in Figure 7). The tighter the fit of the model onto the main peak of the distribution the better the model describes true differences in form.

Finally, for the three types of assessment (abrupt changes, asymmetry and discordancy) in Figure 4, the Ext-P ML-estimator is used with two different values of Kappa; *κ *= 2 (robust superimposition) and *κ *= 6 (un-weighted superimposition, mimicking the Procrustes LSS superimposition (see Figure 7)). Histograms of pooled X, Y, and Z directed local form differences after superimposition are visualized in Figure 8, with the respective estimated AWGN models overlaid. The tighter the model-fit onto the histogram, the better the respective model assumption and therefore the better the superimposition. For all three assessments, the robust superimposition with *κ *= 2, provides a better model in the case of a presented facial abnormality.

## Competing interests

The authors declare that they have no competing interests.

## Authors' contributions

PC, DV and PS devised the technical methods and details. PC, MW and JC explored the biomedical applications, re-worked and applied the methodology on all the cases. MW collected the clinical and required facial data to build up a population norm. PC, DV and JC situated everything within morphometrics and defined the concept of dysmorphometrics. KD, as part of her master's dissertation, generated the computational results. PC wrote the manuscript with input and revisions from KD, MW, JC, DV and PS. All authors have read and approved the final manuscript.
